# Phenotype and molecular characterization of K1-ST700 hypervirulent *Klebsiella pneumoniae* isolated from bone marrow aspirate of a patient with multi-system infection

**DOI:** 10.1186/s12866-026-05068-7

**Published:** 2026-06-09

**Authors:** Chunhong Shao, Xia Chen, Tingchao Wu, Wenyang Wang, Yan Wang, Meijie Jiang

**Affiliations:** 1 Department of Laboratory Medicine, Shandong Provincial Hospital Affiliated to Shandong First Medical University, 324 Jingwu Road, Jinan, Shandong 250021 China; 2 Emergency Department, The Affiliated Taian City Central Hospital of Qingdao University, 29 Longtan Road, Tai’an, Shandong 271000 China; 3 Department of Laboratory Medicine, The Affiliated Taian City Central Hospital of Qingdao University, 29 Longtan Road, Tai’an, Shandong 271000 China; 4 Hematology Department, The Affiliated Taian City Central Hospital of Qingdao University, 29 Longtan Road, Tai’an, Shandong 271000 China

**Keywords:** *K. pneumoniae*, Hypervirulent, Bone Marrow Aspirate, K1, ST700

## Abstract

**Background:**

Worldwide, hypervirulent *Klebsiella. pneumoniae* (HvKp) is a leading cause of multisystem infection. However, it is very rare for HvKp to be cultured from bone marrow aspirates. In this study, four K1-ST700 *Klebsiella pneumoniae* (*K. pneumoniae*) isolates were acquired from the bone marrow, blood, liver abscess puncture fluid, and bronchoalveolar lavage fluid (BALF) of a 53-year-old male patient in a Chinese hospital. The patient rapidly developed fatal multiple-organ failure caused by K1-ST700 HvKp infection.

**Methods:**

Isolates were determined as *K. pneumoniae* via the VITEK-2 Compact system and validated by the VITEK-MS system. Antimicrobial susceptibility assessment of four isolates was conducted via the VITEK-2 Compact system. MLST of *K. pneumoniae* was conducted following the Pasteur Institute protocol. The virulence of isolates was detected through serotype and mouse lethality assay. The bone marrow isolate (KPN-BM) underwent whole-genome sequencing and was compared to available K1 *K. pneumoniae* genomes from China.

**Results:**

All isolates exhibited high virulence in a mouse infection model and remained susceptible to all tested antibiotics. The complete genome sequence was deposited in GenBank (accession no. JBRNXP000000000). A total of 133 virulence-associated genes were identified in KPN-BM, including genes involved in iron acquisition, immune evasion, bacterial adherence, and type VI secretion. A total of 64 *K. pneumoniae* isolates of K1 reported in China exhibited stable phylogenetic relationships. Fifty-eight strains belong to the ST23 type. Five K1-ST700 *K. pneumoniae* strains from China and Norway carry similar virulence genes.

**Conclusions:**

K1-ST700 HvKp may represent an emerging high virulence clone with the potential for transmission. HvKp-induced bone marrow infection is very rare and should be given clinical attention.

**Supplementary Information:**

The online version contains supplementary material available at 10.1186/s12866-026-05068-7.

## Background

*Klebsiella pneumoniae* (*K. pneumoniae*) is one of the important iatrogenic infectious bacteria. Recently, the emergence of hypervirulent *K. pneumoniae* (HvKp) and carbapenem-resistant *K. pneumoniae* (CRKp) has made this bacterium a key threat to public health [1]. In 1986, a community-acquired liver abscess caused by HvKp was first reported in Taiwan, China. Subsequently, it has been reported in the Asia Pacific region and globally [2, 3]. With the research on severe infections, HvKp has gradually received widespread attention. Compared to classic *K. pneumoniae* (cKp), HvKp exhibits stronger virulence characteristics and adaptability, and can not only spread in healthy populations but also lead to life-threatening invasive infections [4]. According to previous research, the 30-day all-cause mortality rate of HvKp-infected patients is 28.4%. The mortality rate of invasive infections is 42.0% [5, 6].

Clinical features, capsule typing, the hypermucoviscous (HMV) phenotype, and the existence of virulence-correlated genes can be utilized to distinguish HvKp from cKp strains [7]. The capsule polysaccharide is a key virulence factor that enhances resistance to phagocytosis and serum killing. A total of 186 KL types have been identified through comparative genomics, and additional K loci are expected as more *K. pneumoniae* genomes become available. Notably, the K1 and K2 serotypes account for over 70% of reported HvKp infections worldwide [8]. More than half of patients with liver abscesses can develop invasive infections, leading to severe damage to multiple tissues and organs such as the lungs, eyes, fascia, central nervous system, and even endangering life [4]. However, it is very rare for HvKp to be cultured from bone marrow aspirates [9]. Herein, a K1-ST700 *K. pneumoniae* was isolated from the bone marrow aspirate of a patient suffering from a multiple-system infection in a teaching hospital, China. The genotypic and phenotypic characteristics of the isolate were characterized.

## Materials and methods

### Bacterial strains

Four strains of *K. pneumoniae* were extracted from bone marrow, blood cultures, a liver abscess puncture fluid, and BALF of a 53-year-old man admitted to the hematology department of a teaching hospital in China on August 29, 2023. Two days before admission, the patient developed a fever after catching a cold, with a maximum temperature of 39.5 °C, along with symptoms (fatigue, cough, phlegm, dizziness, palpitations, and chest tightness). The patient had a > 20-year history of type 2 diabetes and no history of international travel. Informed consent was obtained, and clinical data were retrieved from electronic medical records. The investigation received approval from the Ethics Committee of Taian City Central Hospital affiliated with Qingdao University (Approval Number: 2025-08-D40), and was conducted as per institutional guidelines. Isolates were determined as *K. pneumoniae* via the VITEK-2 Compact system and validated by the VITEK-MS system (BioMérieux, France). Four *K. pneumoniae* extracted from bone marrow, blood, liver abscess puncture fluid, and BALF were designated as KPN-BM, KPN-BL, KPN-AB, and KPN-BA.

### HMV phenotype and serotype analysis

The HMV phenotype was assessed using the string test. Colonies grown overnight on blood agar at 37 °C were stretched with a bacteriology loop, and a viscous string > 5 mm indicated a positive result. Serotyping for K1, K2, K5, K20, K54, and K57 was performed as previously described [10].

### Antibiotic susceptibility assessment

Antimicrobial susceptibility assessment was conducted via the VITEK-2 Compact system (BioMérieux, France). The minimum inhibitory concentrations (MICs) of imipenem, meropenem, and ertapenem were detected by E-test (BioMérieux, France). *E. coli* ATCC 25922 and *K. pneumoniae* ATCC 700603 were utilized as quality controls. All outcomes were interpreted according to the 2025 EUCAST breakpoints.

### Mouse lethality assay

To detect the virulence of four *K. pneumoniae* isolates, we utilized pathogen-free, 6–8 weeks-old, male C57BL/6 mice [Changzhou Cavion Experimental Animal Co, Ltd. (license no. SCXY (Su) 2011-0003)]. Herein, for each bacterial concentration, six mice were utilized. *K. pneumoniae* ATCC 13883 was considered the control strain. An intraperitoneal injection of 0.1 mL of a 10⁵CFU bacterial suspension was introduced into C57BL/6 mice, and symptoms and death were monitored for ten days. We performed a log-rank (Mantel–Cox) test to compare the survival curves of mice infected with the four clinical isolates versus the control strain. Liver, lung, and brain tissues were subsequently collected for pathological examination.

### Histopathological examination and semi-quantitative scoring

Tissue samples were fixed in 10% neutral buffered formalin, embedded in paraffin, sectioned at 4–5 μm thickness, and stained with hematoxylin and eosin (H&E) for histopathological evaluation. All slides were examined under a light microscope by two independent pathologists who were blinded to the experimental groups. A semi-quantitative scoring system (SQS) was established based on previously described methods for murine models of acute hepatitis, pneumonia, and encephalitis/meningitis [11]. For each organ, three morphological parameters were assessed and graded on a scale from 0 to 3, where higher scores indicate more severe pathology. Histopathological scores are presented as median (range). Comparisons between the KPN-BM infected group and the control group were performed using the Mann-Whitney U test for each parameter. Exact p-values were calculated. A p-value < 0.05 was considered statistically significant for the primary outcome measures.

### Multilocus sequence typing (MLST)

MLST of *K. pneumoniae* was conducted following the Pasteur Institute protocol. Seven housekeeping genes (gapA, infB, mdh, pgi, phoE, rpoB, and tonB) were amplified, sequenced, and compared to alleles in the MLST database.

### Genomic analysis

The draft genome of KPN-BM was generated by Shanghai Biozeron Biotechnology (Shanghai, China). Sequencing was conducted on the Illumina HiSeq Xten platform (San Diego, U.S) using a TruSeq Nano DNA LT shotgun library prepared for paired-end sequencing. Raw reads were produced with CASAVA v1.8.23 and assembled using SOAP denovo. Annotation of contigs longer than 500 bp was conducted via the National Center for Biotechnology Information (NCBI) Prokaryotic Genome Annotation Pipeline (PGAP) and the Rapid Annotation using Subsystem Technology (RAST) server (http://rast.nmpdr.org/). Metabolic pathways were inferred by annotating the genomic sequences against the Kyoto Encyclopedia of Genes and Genomes (KEGG) database (https://www.genome.jp/kegg/pathway.html). The prediction of virulence and pathogen–host interaction (PHI) factors was conducted using the Virulence Factor Database (VFDB) (http://www.mgc.ac.cn/VFs/main.htm), and the virulence factors of KPN-BM were compared with other four additional K1-ST700 *K. pneumoniae* strains.

### Construction of a phylogenetic tree

The NCBI database was utilized to retrieve the Genomes of *K. pneumoniae* with capsular genotype K1 using the query “K1, *K. pneumoniae*,” yielding 601 genomes, including 63 from China. A maximum likelihood phylogenetic tree was created via PhyML v3.0 (http://www.atgc-montpellier.fr/phyml/) with 1000 bootstrap replicates.

## Results

### Clinical characteristics

**Fig. 1:**
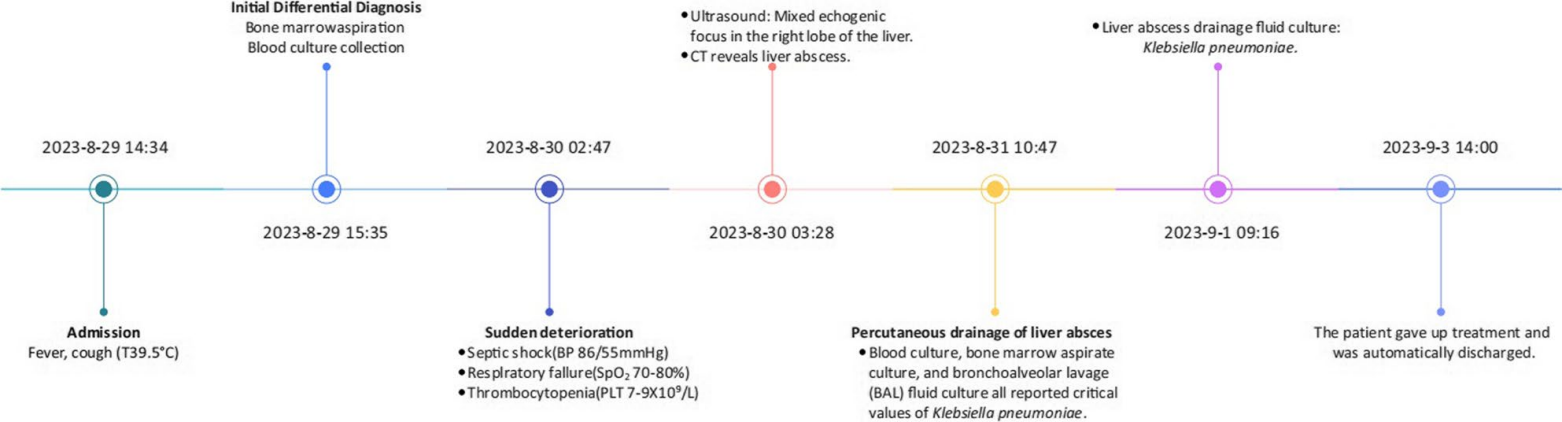
Clinical characteristics and treatment process of the patient

The patient was admitted with a fever (39.5 °C), heart rate 87/min, respiration 18/min, and blood pressure 99/59 mmHg. Laboratory examinations illustrated elevated leukocytosis with neutrophilia (9.44 × 10^9^ cells/L with 93.1% neutrophils), platelet count 7 × 10^9^ cells/L, blood glucose 29.70 mmol/L, and procalcitonin (PCT, 17.83 mg/L, normal value: < 0.5 mg/L). Upon initial chest CT, multiple nodules and patchy lesions were observed in both lungs, particularly in the subpleural region, with some lesions exhibiting “melted ice” changes and intralesional vascular connections, suggesting septic pulmonary embolism. A slightly hypodense lesion resembling a round shape was detected in the right lobe of the liver, possibly indicating a liver abscess. The following thoracoabdominal CT revealed a significant increase in the lesion’s extent, with extensive consolidation in the lower lobes of both lungs. Gas density was observed within the hypodense area of the right lobe of the liver, consistent with the appearance of a liver abscess (supplementary Figure S1). Due to the severity of the patient’s condition, he were transferred to the emergency ICU ward on August 30, 2023, and underwent ventilator-assisted breathing. From August 31st to September 2nd, four strains of *K.pneumoniae* were extracted from patients’ bone marrow, blood, liver abscess puncture fluid, and BALF (Fig. 1).

The patient was exposed to meropenem (2 g/8 h), levofloxacin (0.5 g/24 h), and received plasma and platelet transfusions for treatment. He also underwent liver abscess puncture and drainage surgery on September 1, 2023. Unfortunately, the patient gave up treatment and was automatically discharged on September 3, 2023. A follow-up was conducted afterwards, and the patient ultimately died.

### Drug sensitivity and MLST of four *K. pneumoniae* isolates

The antimicrobial susceptibility testing of four isolates of *K. pneumoniae* from different specimens are completely identical, except for resistance to trimethoprim/sulfamethoxazole; they were susceptible to other tested antibiotics (e.g., aztreonam, carbapenems, ampicillin/sulbactam, piperacillin/tazobactam, cephalosporins, quinolones, and aminoglycosides) (supplementary Table S1). MLST illustrated that the sequence type of four *K. pneumoniae* isolates was ST700.

### Virulence of four *K. pneumoniae* isolates

**Fig. 2:**
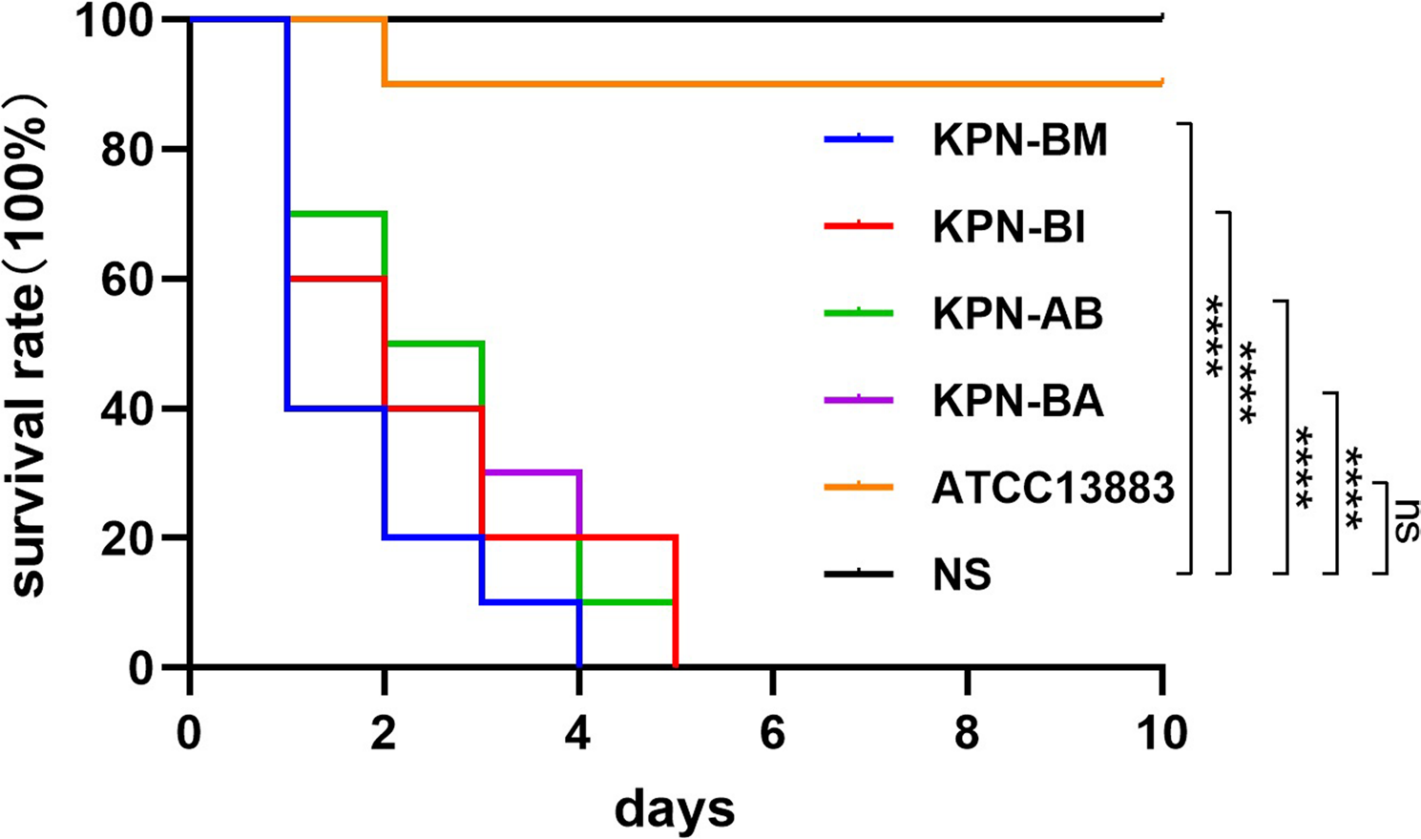
The survival rate of mouse infected by four HvKp isolates. *K. pneumoniae* ATCC 13883 and normal saline (NS) served as the control. Statistical significance: ****, *P* < 0.0001;ns, not significant

The string test confirmed HMV in all four isolates, and PCR identified their K1 serotype. In the mouse infection model, these isolates exhibited hypervirulence comparable to *K. pneumoniae* ATCC 13883. Mean mouse survival rates were 60% on day 1 and 20% on day 3, with complete mortality by day 5 for all clinical isolate infected groups. In contrast, ATCC 13883-infected mice showed only 10% mortality (Fig. 2). The results showed statistically significant differences in survival distributions (*P* < 0.001), confirming the hypervirulent nature of the clinical isolates.

**Fig. 3:**
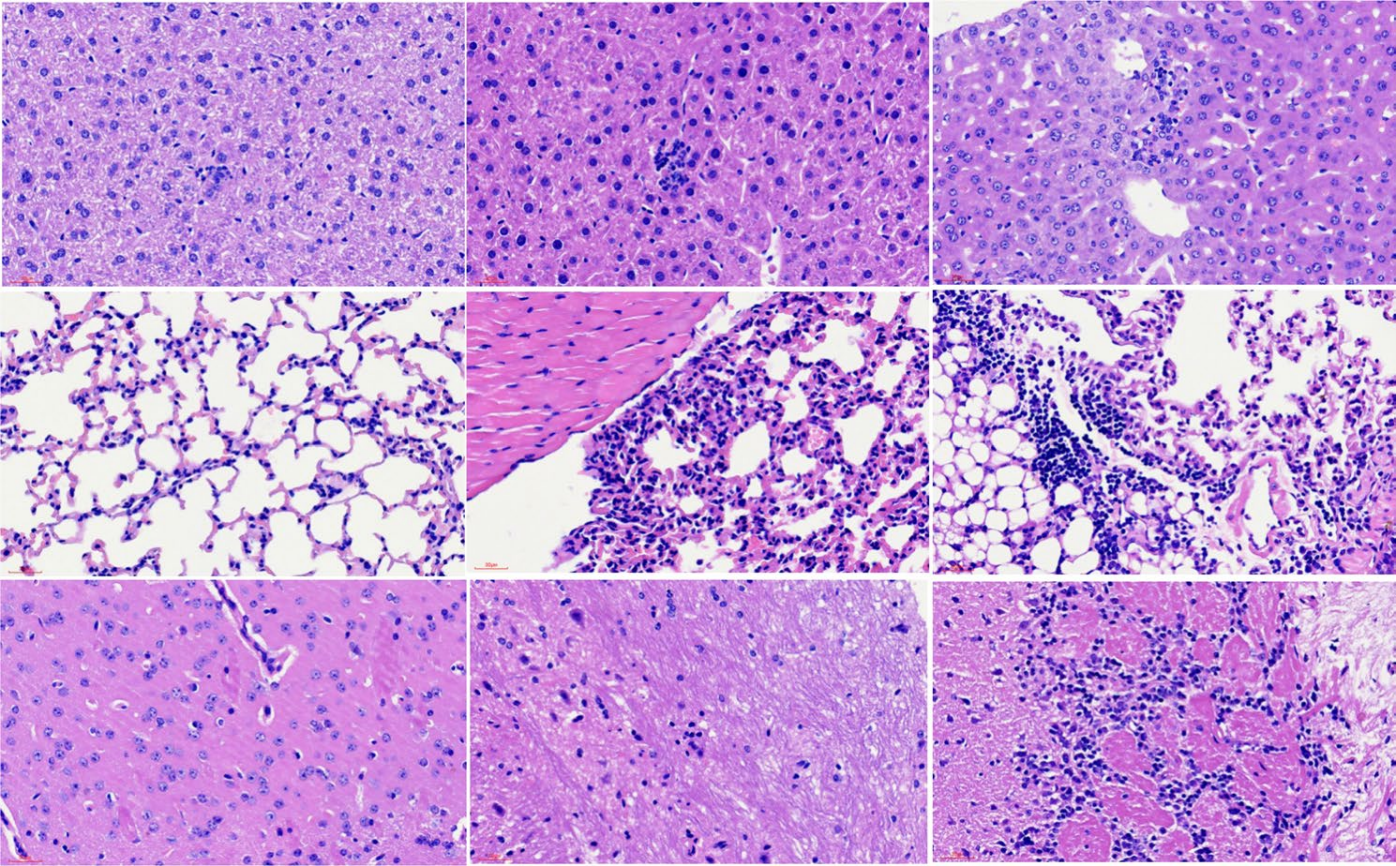
Histopathological changes of normal tissues and HvKp-infected tissues in mice. The three images on the left represent normal liver, lung, and brain tissues of mice, respectively. The three images in the middle represent liver, lung, and brain tissues of mice after ATCC13883 infection, respectively.The three images on the right represent liver, lung, and brain tissues of mice after HvKP-BM infection, respectively

The pathological results showed that the tissues of mice injected with saline were normal, and no inflammatory response was observed. Mild inflammatory reactions were observed in tissues from different parts infected with *K. pneumoniae* ATCC 13883, with mild proliferation of alveolar epithelial cells and glial cells, and scattered lymphocyte infiltration in the interstitium. However, mice infected with clinical isolates showed significant inflammatory reactions in all three locations. The liver cells were large in volume and polygonal in shape, with a circular nucleus located in the center and visible nucleoli. The cytoplasm was pink in color, and there was multifocal infiltration of lymphatic monocytes in the interstitium. The main pathological changes in lung tissue are alveolar epithelial hyperplasia and chronic inflammatory cell infiltration in the interstitium. The main pathological changes in brain tissue are chronic inflammatory cell infiltration within the brain parenchyma (Fig. 3). The histopathological scores of liver, lung, and brain tissues from mice infected with KPN-BM and ATCC13883 also exhibited significant difference (Supplementary Table S2).

### Genomic characterization of KPN-BM

The draft genome of KPN-BM measured 5,222,405 bp with a GC content of 51.69%. A total of 5,191 protein-coding genes were annotated. KEGG analysis detected 3,520 genes distributed across 20 biological functions. KPN-BM harbored a few antibiotic resistance genes, consistent with its classification as an HvKp pathotype and in agreement with its antibiotic susceptibility phenotype.

**Fig. 4:**
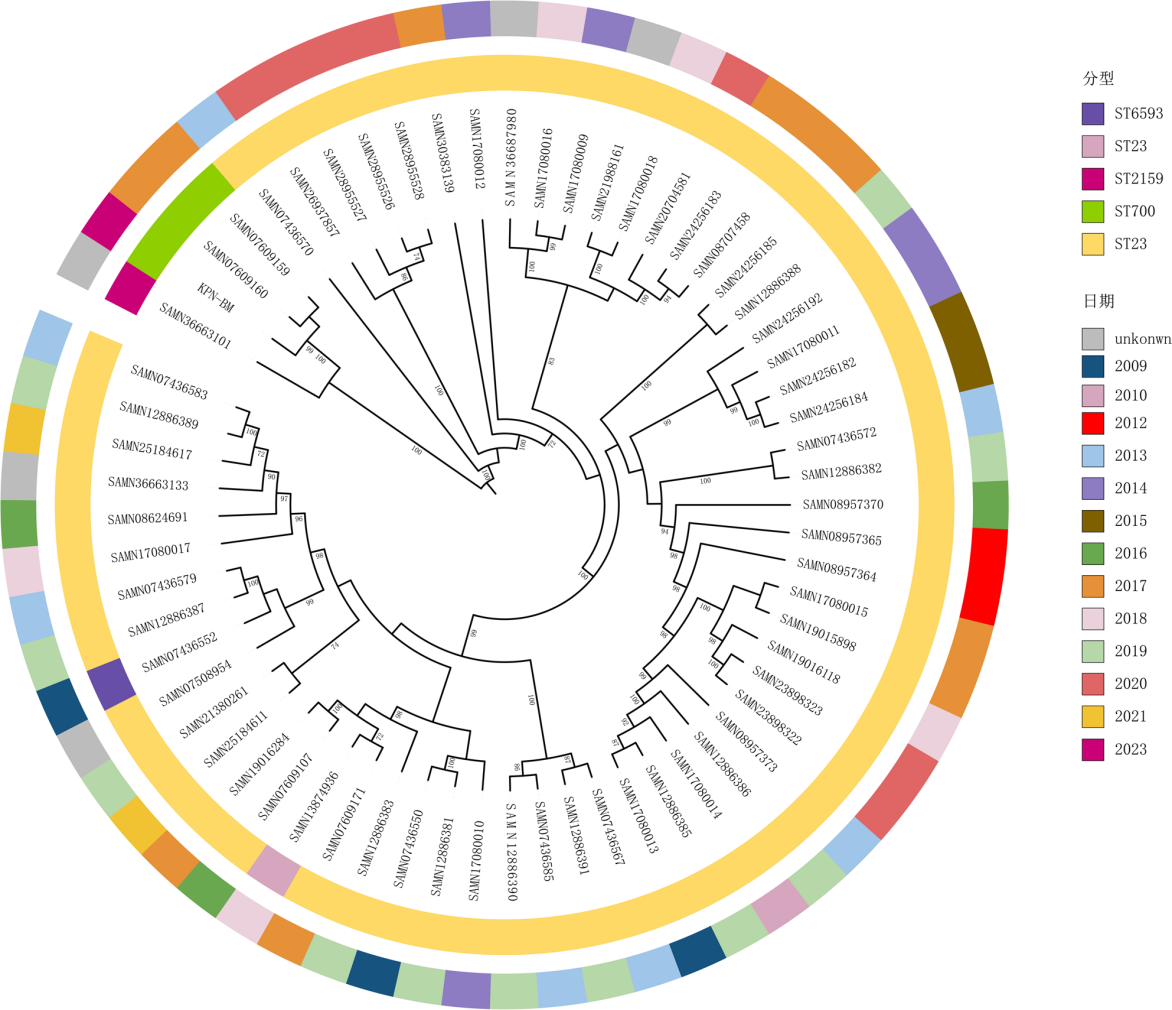
A phylogenetic tree of 64 K1-HvKp isolates from China. Note: the numbers on the branch indicate the branch reliability, The closer the value is to 100, the higher the reliability is. The branch length represents the evolutionary distance, which is calculated by the average number of substitutions per nucleotide

The complete sequence of KPN-BM was submitted to GenBank (accession no. JBRNXP000000000). The full published sequences of 63 *K. pneumoniae* of K1 from China were downloaded, and a phylogenetic tree was constructed. Since 2009, the 64 *K. pneumoniae* isolates of serotype K1 reported in China exhibited remarkably stable phylogenetic relationships, with bootstrap values consistently exceeding 70%. A total of 58 strains belong to the ST23 type. SAMN07609159 and SAMN07609160 belong to ST700 (Fig. 4).

### Identification of virulent and PHI factors

**Fig. 5:**
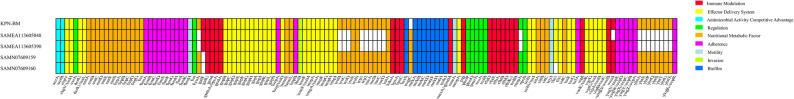
Bacterial virulence factors analysis of KPN-BM and 4 other K1-ST700 *K.pneumoniae*

With an identity ≥ 70%, a total of 145 virulence-related genes and 533 pathogen-correlated genes were predicted via the VFDB and the PHI database, respectively. Among the 145 virulence genes, 41 genes are involved in iron uptake, 35 genes are related to immune evasion function, 26 genes are engaged in bacterial adherence, and 18 genes are related to the type VI secretion system. In addition, the bacterial virulence factors of KPN-BM were compared with those of four other K1-ST700 *K. pneumoniae*. SAMN07609159 and SAMN07609160 were reported in Sichuan province, China. SAMEA113605048 and SAMEA1136053902017 were from Norway. The outcomes illustrated that most virulence genes were present in all five isolates simultaneously. However, there are 19 virulence genes in KPN-BM, but they are missing in SAMEA113605048 and SAMEA113605390 from Norway, which are mainly involved in iron uptake and regulation, including *rmpA*. Only two genes showed differences between KPN-BM compared with SAMN07609159 and SAMN07609160 from China. Gene *galF* is related to immune regulation, and *tle1* belongs to the effectiveness delivery system (Fig. 5).

## Discussion

HvKp typically results in pyogenic liver abscesses acquired from the community in individuals without prior clinical history [12]. It is also a major cause of multisystem infections, including pneumonia, hepatic and extrahepatic abscesses, endophthalmitis, meningitis, soft-tissue infections, and necrotizing fasciitis [13, 14]. However, bone marrow infections caused by HvKp are extremely rare, as the pathogen must overcome the physical barriers of skin and mucous and the robust blood defense system to invade the bone marrow. Moreover, the bone marrow, serving as the origin and residence of immune cells, possesses formidable internal defenses. The intricate structure and blood flow within the bone marrow make it difficult for bacteria to establish an infection. Once bacteria take root in the bone marrow, they will trigger severe inflammatory reactions, leading to prolonged infections that are highly difficult to treatment [15, 16]. Due to unexplained severe and persistent thrombocytopenia of this patient, we collected his bone marrow for culture to assess whether there was sepsis-related bone marrow suppression. Bone marrow, as a relatively sterile tissue, provides strong direct evidence for systemic hematogenous dissemination of pathogens when its culture results match those in blood and abscess samples. Diabetes mellitus is a major risk factor for HvKp infection, which predominantly affects males aged 50–60 years [17, 18]. In this case, the 53-year-old diabetic patient exhibited clinical features consistent with previous reports. The clinical course showed hallmark characteristics of HvKp infection [19]: severe disease with bone marrow invasion from the bloodstream, involvement of multiple sites, including the respiratory system and liver abscess, and rapid, notably aggressive progression.

The predominant circulating HvKp serotypes comprise K1, K2, K20, K54, and K57, with K1 and K2 representing the most infectious types and comprising approximately 70% of isolates [20]. Whole-genome sequencing provides high-resolution clonal group classifications, enabling clear differentiation of *K. pneumoniae* lineages. To investigate the evolutionary relationships of K1 *K. pneumoniae* in China, nucleotide sequences from 63 K1 HvKp isolates were retrieved and compared with KPN-BM. ST23 emerged as the dominant subtype, while only two isolates belonged to ST700, consistent with previous reports [21, 22]. ST23 shows a strong association with the K1 capsular type and is typically linked to four siderophore-mediated iron acquisition systems, the colibactin, and highly invasive infections [23]. There are two other HvKp strains of K1-ST700 in the database, but no specific reports have been found. K1-ST23 hypervirulent *K.pneumoniae* possesses a more complete virulence gene system. The genome sequencing results of the KPN-BM in this study lack part of colibactin. Furthermore, carbapenem-resistant K1-ST23 *K.pneumoniae* has been widely reported [24], whereas carbapenem-resistant K1-ST700 isolates have not been found yet. Due to the limited number of strains, it is currently unclear whether ST700 is highly related to the K1 capsular type. It indicates that the K1-ST700 HvKp may represent an emerging high-virulence clone with the potential for successful transmission.

Except for capsule types, several other factors of HvKp are credited to its virulence and pathogenesis, comprising up to four siderophore systems for iron acquisition, the type I and III fimbriae, the colibactin toxin, and the type VI secretion system [25]. The bacterial virulence factors of KPN-BM were compared to those of the other four K1-ST700 HvKp. The outcomes illustrated that three HvKp from China had the almost same virulence factors. Nineteen virulence factors were missed in two K1-ST700 HvKp from Norway, including *rmpA* and 18 genes (e.g., *iucA*, *iroB*) related to iron acquisition. Among the 19 genes, only the four genes (*iucABCD*) encoding aerobactin are located on the plasmid. Other 15 genes, including *rmpA*, are located on the chromosome of KPN-BM. These genes are primarily related to iron metabolism and mucoid phenotype regulation. The association between rmpA and HMV is extremely high, and the existence of *rmpA* among a set of genes has been considered a biomarker to detect potential HvKp strains [26]. At the same time, it indicates that the acquisition and accumulation of iron carrier systems are key factors to increase the toxicity and invading of K1-ST700 HvKp. Thus, to counteract host nutritional immunity, invading bacterial pathogens encode high-affinity iron acquisition systems [27]. In addition, type VI system (T6SS) can promote bacterial competition, cell invasion, type-1 fimbriae expression, and in vivo colonization in *K. pneumoniae* [28]. KPN-BM carried 18 related genes. Previous studies have shown that *K. pneumoniae* producing abscesses have an increased incidence of T6SS genes, and T6SS-positive *K. pneumoniae* was common in people who had BSIs [29, 30]. This indicates that T6SS may participate in the HvKp virulence mechanism and bacterial competition.

Although early HvKp isolates were generally antimicrobial-susceptible, effective management still requires rapid therapeutic intervention to prevent dissemination. In this case, all four HvKp isolates were susceptible to the tested antibiotics, and meropenem was promptly administered due to the multisite infections. However, the patient did not survive. This highlights the need for advanced diagnostics to facilitate rapid identification of HvKp in clinical settings. Given the diversity of STs, capsule types, and the existence of HMV in HvKp isolates, accurate and timely identification is critical to guide appropriate treatment and improve patient outcomes [31].

## Conclusions

In brief, our investigation illustrated the phenotype and molecular characteristics of K1-ST700 HvKp, which causes multi-system infection in individuals with type 2 diabetes. K1-ST700 HvKp may represent an emerging high virulence clone with the potential for transmission. HvKp-induced bone marrow infection is very rare and should be given clinical attention. Additional epidemiological investigations are needed to clarify the virulence factors, pathophysiology, and clinical characteristics related to the K1-ST700 HvKp strain infection.

## Supplementary Information

Supplementary Material 1.

Supplementary Material 2.

Supplementary Material 3.

## Data Availability

All data generated or analyzed during this study are included in this published article and its supplementary information files. The whole-genome sequencing data were archived in the NCBI, with strain KPN-BM designated with the SRA accession no. JBRNXP000000000 (https://www.ncbi.nlm.nih.gov/gene/?term=JBRNXP000000000).
