# Supplementary material for: Phenotype and molecular characterization of K1-ST700 hypervirulent Klebsiella pneumoniae isolated from bone marrow aspirate of a patient with multi-system infection
Source: BMC Microbiol. 2026 Jun 9;26:726. doi: 10.1186/s12866-026-05068-7 (PMC13474677; doi:10.1186/s12866-026-05068-7)
Supplement: Supplementary file 1 [file 12866_2026_5068_MOESM3_ESM.docx]

Supplementary Table S2

**Histopathological scores of liver, lung, and brain tissues in infected mice**

| **Lung** | Alveolar wall inflammation | Perivascular/peribronchial infiltration | Intra-alveolar neutrophils | Edema/hemorrhage | **Total Score** |
| --- | --- | --- | --- | --- | --- |
| KPN-BM | 3 (2–3) | 2（2-3） | 2（2-3） | 2（1-2） | **9（7-11）** |
| Control | 0（0-1） | 0（0-0） | 0（0-0） | 0（0-0） | **0（0-1）** |
| *p-value* | *0.008* | *0.046* | *0.046* | 0.046 | *0.008* |
| **Liver** | Inflammatory Infiltration | Tissue Damage | Structural Alteration | / | **Total Score** |
| KPN-BM | 3（2-3） | 2（2-3） | 2（2-3） | / | **7（6-9）** |
| Control | 0 (0–0) | 0 (0–0) | 0 (0–0) | / | **0 (0–0)** |
| *p-value* | *0.008* | *0.008* | *0.008* | / | *0.008* |
| **Brain** | Meningeal inflammation | Perivascular cuffing | Parenchymal infiltration | Neuronal necrosis | **Total Score** |
| KPN-BM | 3（2-3） | 2（2-3） | 2（1-2） | **2（1-2）** | **9（7-10）** |
| Control | 0 (0–0) | 0 (0–0) | 0 (0–0) | **0 (0–0)** | **0 (0–0)** |
| *p-value* | *0.008* | *0.008* | *0.008* | *0.008* | *0.008* |
