# Supplementary material for: Phenotype and molecular characterization of K1-ST700 hypervirulent Klebsiella pneumoniae isolated from bone marrow aspirate of a patient with multi-system infection
Source: BMC Microbiol. 2026 Jun 9;26:726. doi: 10.1186/s12866-026-05068-7 (PMC13474677; doi:10.1186/s12866-026-05068-7)
Supplement: Supplementary file 2 [file 12866_2026_5068_MOESM2_ESM.doc]

Supplementary Table S1 Antibiotic susceptibilities of four *K. pneumoniae* isolates (μg/ml).

SAM, ampicillin sulbactam; TZP, piperacillin/tazobactam; ATM, aztreonam; CRO, cefatriaxone; CAZ, ceftazidime; FEP, cefepime; IPM, imipenem; MEM, meropenem; ETP, ertapenem; CN, gentamicin; AK, amikacin; CIP, ciprfloxacin; LEV, levofloxacin; STX, trimethoprim/sulfamethoxazole.

| Isolates | SAM | TZP | ATM | CRO | CAZ | FEP | IMP | MEM | ETP | CN | AK | CIP | LEV | SXT |
| --- | --- | --- | --- | --- | --- | --- | --- | --- | --- | --- | --- | --- | --- | --- |
| TAKPN-BM | <=2/1 | <=4/4 | <=1 | <=1 | <=1 | <=1 | <=0.02 | ≤0.02 | ≤0.02 | <=1 | <=2 | ≤0.25 | ≤0.25 | >2/38 |
| TAKPN-BL | <=2/1 | <=4/4 | <=1 | <=1 | <=1 | <=1 | <=0.02 | ≤0.02 | ≤0.02 | <=1 | <=2 | ≤0.25 | ≤0.25 | >2/38 |
| TAKPN-AB | <=2/1 | <=4/4 | <=1 | <=1 | <=1 | <=1 | <=0.02 | ≤0.02 | ≤0.02 | <=1 | <=2 | ≤0.25 | ≤0.25 | >2/38 |
| TAKPN-BA | <=2/1 | <=4/4 | <=1 | <=1 | <=1 | <=1 | <=0.02 | ≤0.02 | ≤0.02 | <=1 | <=2 | ≤0.25 | ≤0.25 | >2/38 |
